# Metabolic benefits of 1-(3-(4-(*o*-tolyl)piperazin-1-yl)propyl)pyrrolidin-2-one: a non-selective α-adrenoceptor antagonist

**DOI:** 10.1007/s40618-017-0779-7

**Published:** 2017-11-07

**Authors:** Magdalena Kotańska, Katarzyna Kulig, Monika Marcinkowska, Marek Bednarski, Katarzyna Malawska, Paula Zaręba

**Affiliations:** 1 Department of Pharmacodynamics, Jagiellonian University Medical College, 9 Medyczna Street, 30-688 Kraków, Poland; 2 Chair of Pharmaceutical Chemistry, Department of Physicochemical Drug Analysis, Faculty of Pharmacy, Jagiellonian University Medical College, Medyczna 9, 30-688 Kraków, Poland; 3 Chair of Pharmaceutical Chemistry, Department of Medicinal Chemistry, Faculty of Pharmacy, Jagiellonian University Medical College, Medyczna 9, 30-688 Kraków, Poland; 4 Department of Pharmacological Screening, Jagiellonian University Medical College, 9 Medyczna Street, 30-688 Kraków, Poland

**Keywords:** α-Adrenoceptor antagonist, Hyperglycemia, Metabolic disorders, Pyrrolidin-2-one derivative

## Abstract

**Purpose:**

Previous studies have shown that several components of the metabolic syndrome, such as hypertension, obesity or imbalanced lipid and carbohydrate homeostasis, are associated with the sympathetic nervous system overactivity. Therefore, the inhibition of the adrenergic nervous system seems to be a reasonable and appropriate therapeutic approach for the treatment of metabolic disturbances. It has been suggested that non-selective adrenoceptor antagonists could be particularly beneficial, since α_1_-adrenoceptor antagonists can improve disrupted lipid and carbohydrate profiles, while the inhibition of the α_2_-adrenoceptor may contribute to body weight reduction. The aim of the present study was to investigate the metabolic benefits deriving from administration of a non-selective α-adrenoceptor antagonist from the group of pyrrolidin-2-one derivatives. The aim of the present study was to investigate the potential metabolic benefits deriving from chronic administration of a non-selective α-adrenoceptor antagonist, from the group of pyrrolidin-2-one derivatives.

**Methods:**

The α_1_- and α_2_-adrenoreceptor affinities of the tested compound—1-(3-(4-(*o*-tolyl)piperazin-1-yl)propyl)pyrrolidin-2-one had been investigated previously by means of the radioligand binding assay. In the present study, we extended the pharmacological profile characteristics of the selected molecule by additional intrinistic activity assays. Next, we investigated the influence of the tested compound on body weight, hyperglycemia, hypertriglyceridemia, blood pressure in the animal model of obesity induced by a high-fat diet, and additionally we measured the spontaneous activity and body temperature.

**Results:**

The intrinistic activity studies revealed that the tested compound is a potent, non-selective antagonist of α_1B_ and α_2A_-adrenoceptors. After the chronic administration of the tested compound, we observed reduced level of triglycerides and glucose in the rat plasma. Interestingly, the tested did not reduce the body weight and did not influence the blood pressure in normotensive animals. Additionally, the administration of the tested compound did not change the animals’ spontaneous activity and body temperature.

**Conclusion:**

Non-selective α-adrenoceptor antagonist seems to carry potential benefits in the improvement of the reduction of elevated glucose and triglyceride level. The lack of influence on blood pressure suggests that compounds with such a pharmacological profile may be particulary beneficial for the patients with disturbed lipid and carbohydrate profile, who do not suffer from hypertension. These results are particulary valuable, since currently there are no safe α_2A_-adrenoceptor antagonist drugs available in clinical use with the ability to modulate hyperglycemia that would not affect blood pressure.

## Introduction

The metabolic syndrome has been defined as a simultaneous occurrence of at least three out of five symptoms, such as impaired fasting glycaemia, abdominal obesity, elevated blood pressure, hypertriglyceridemia, and low levels of high density lipoprotein (HDL) [1]. The metabolic syndrome significantly increases the risk of developing serious conditions, such as diabetes, coronary heart disease, ischemic stroke and artheroscleroris. Recent statistic reports have shown that the metabolic syndrome occurs in 20-25% of the adult population, and it has already reached epidemic proportions [2]. It constitutes a serious therapeutical challenge for the health care system in developed countries, and consequently there has been a great emphasis on developing effective and safe therapies.

Previous studies have revealed that several components of the metabolic syndrome are associated with an increased activation of the sympathetic nervous system. For instance, the adrenergic nervous system plays a key role in the induction and progression of high blood pressure, alteration of the cardiac output, peripheral vascular resistance as well as controlling the triglyceride and glucose metabolism [3]. It has been shown that the sympathetic nerve activation is much higher in patients with metabolic syndrome, comparing with healthy subjects [4]. Moreover, when hypertension and obesity are present simultaneously, the sympathetic activation is significantly higher comparing with patients with a single disease [5]. Therefore, the inhibition of the adrenergic nervous system constitutes a suitable therapeutic approach for the treatment of the components of the metabolic syndrome [6].

The α-adrenoceptors seem to be an interesting molecular target, since many of the undesirable reactions are mediated via a stimulation of α-adrenoceptors, such as vasoconstriction, and a release of free fatty acids and glucose. It has been postulated that particularly non-selective adrenoceptor antagonists may be beneficial for patients with metabolic syndrome [7]. The inactivation of α_1_-adrenoceptors causes vasodilatation and results in the reduction of blood pressure [8]. Moreover, the α_1_-adrenoceptor antagonists exert a positive effect on the lipid and carbohydrate profiles [9, 10]. The blockade of α_2_-adrenoceptors can lead to the reduction of body weight due to the activation of catecholamine release, sympathetic nervous system stimulation, and induction of lipolysis and thermogenesis [11]. It was also reported that the α_2_-adrenoceptor antagonists might favorably modulate the pancreatic function and abnormal insulin secretion [12].

Over the past few years, an extensive research on the pyrrolidin-2-one derivatives and its pharmacological activities has been conducted. The structure activity relationship within this group of compounds revealed that they possess a high affinity for the α-adrenoceptors and exert antagonistic activity [13–16]. So far, the pyrrolidin-2-one derivatives demonstrated antiarrhythmic and antihypertensive properties [8, 17] and have been reported to reduce the body weight in the animal models of obesity [18].

In the present study, we investigated the effect of the non-selective α-adrenoceptor antagonist; 1-(3-(4-(*o*-tolyl)piperazin-1-yl)propyl)pyrrolidin-2-one (EP-47) on the body weight, hyperglycemia, hypertriglyceridemia, spontaneous activity in animals with obesity induced by a high-fat diet, as well as blood pressure and temperature.

## Materials and methods

### Animals

The experiments were carried out on male Wistar rats (for the obesity studies, the animals’ body weight were 150–160 g, for the blood pressure measurement, animals’ weight were: 220–260 g). The animals were housed in pairs in plastic cages in constant temperature-controlled facilities exposed to a light–dark cycle; water and food were available ad libitum. Control and experimental groups consisted of six to eight animals each. All experiments were conducted in accordance to the guidelines of the Animal Use and Care Committee of the Jagiellonian University and were approved for studies (2012, Poland; Permissions No 54/2012).

### Determination of the intrinsic activity of the tested compound at the α_1A_-adrenoreceptors and α_1B_-adrenoreceptors

The intrinsic activity assay was performed according to the instructions of the manufacturer of the assay kit containing ready-to-use cells with stable expression of the α_1A_-adrenoceptor (Invitrogen, Life Technologies) or α_1B_-adrenoceptor (Perkin Elmer).

### Determination of the intrinsic activity of the tested compound at the α_2A_-adrenoreceptors and α_2B_-adrenoreceptors

The intrinsic activity assay was performed according to the instructions of the manufacturer of the assay kit containing ready-to-use cells with stable expression of the α_2A_-adrenoceptor (Invitrogen, Life Technologies) or α_2B_-adrenoceptor (Perkin Elmer).

### Obesity induced by a fatty diet and influence on body weight

Male Wistar rats (150–160 g) were pair-housed and fed a fatty diet consisting of 40% fat blend (Labofeed B with 40% lard, Morawski, Manufacturer Feed, Poland) for 14 weeks, with water available ad libitum [19, 20]. Control rats were fed a standard diet (Labofeed B, Morawski Manufacturer Feed, Poland).

After 10 weeks, rats with obesity induced via their diet, were randomly divided into three equal groups that had the same mean body weight and were treated intraperitoneally with the test compound at the following doses: 2 or 5 mg/kg b.w./day and control group: vehicle–water 0.3 ml/kg (diet-induced obesity control group) once daily in the mornings between 9:00 a.m. and 10:00 a.m. for 30 days. The rats with obesity (three groups) were maintained on a fatty diet throughout the treatment period (30 days). Control rats (one group) were maintained on a standard diet throughout 30 days, with intraperitoneal administration of vehicle–water.

Fatty feed composition (932 g of dry mass): protein 193 g, fat (lard) 408 g, fiber 28.1 g, crude ash 43.6 g, calcium 9.43 g, phosphorus 5.99 g, sodium 1.76 g, sugar 76 g, magnesium 1.72 g, potassium 7.62 g, manganese 48.7 mg, iodine 0.216 mg, copper 10.8 mg, iron 125 mg, zinc 61.3 mg, cobalt 0.253 mg, selenium 0.304 mg, vitamin A 15,000 units, vitamin D3 1000 units, vitamin E 95.3 mg, vitamin K3 3.0 mg, vitamin B1 8.06 mg, vitamin B2 6.47 mg, vitamin B6 10.3 mg, vitamin B12 0.051 mg, folic acid 2.05 mg, nicotinic acid 73.8 mg, pantothenic acid 19.4 mg, choline 1578 mg.

### Collecting peritoneal fat and the plasma

After the last 13th administration of the test compounds to animals, the feed was discontinued. On the 31st day, 20 min after intraperitoneal administration of heparin 1000 j/rat and 70 mg/kg b.w. thiopental, plasma was collected from the left carotid artery of the animals. The peritoneal fat was removed and weighed.

### The influence of the test compound on lipid and carbohydrate profiles in diet-induced obese rats

To determine the glucose or triglyceride levels in the plasma, standard tests from Biomaxima S.A. (Poland) were used. The substrate was decomposed with the appropriate enzymes for the relevant product, which was converted to a colored compound. Coloration was proportional to their concentration. The absorbance was measured at a wavelength of 500 nm.

### The influence of the tested compound on the locomotor activity after the chronic treatment in diet-induced obese rats housed in pairs in home cages

The locomotor activity of rats treated with the test compound was measured on the 1st, 21st and 29th day of the treatment with a special radio-frequency identification system (RFID)—TraffiCage (TSE-Systems, Germany) [21]. The rats were housed in pairs in home cages, with feed and water available ad libitum. The animals had subcutaneously implanted transmitter identification, which enabled the presence and time spent in different areas of the cage to be recorded, and then the data were collected using a special computer program. The rats were treated intraperitoneally with a compound at a dose of 5 or 2 mg/kg b.w./day or with vehicle–water 0.3 ml/kg (control group) once a day in the mornings for 30 days. The locomotor activity was monitored for 24 h after administration of the test compound.

### Determination of the effect of the tested compounds on blood pressure after a single administration in anesthetized Wistar rats

The rats were anesthetized with thiopental (70 mg/kg) via intraperitoneal injection. The left carotid artery was cannulated with polyethylene tubing filled with heparin solution in saline to facilitate pressure measurements using a PowerLab Apparatus (ADInstruments, Australia). The blood pressure was measured both before intraperitoneal administration of the compound—time 0 min (control pressure) and 90 min thereafter. Pressure was measured in normotensive rats after a single administration of the test compound.

### Determination of the effect of the test compound on the blood pressure during subchronic study in Wistar rats residing in natural housing conditions: telemetric method

The blood pressure of rats treated with the test compound was measured one the 1st and the 15th day, after 5 mg/kg b.w., intraperitoneally treatment with a special telemetric system—Stellar (TSE-Systems, Germany) [18].

*Surgery* The operation was performed over 30 min under sterile conditions. The rats were anesthetized with ketamine and xylasine (intramuscular injection: 100 and 10 mg/kg). Before the surgery and for 7 days after, the animals were additionally treated with cefuroxime (20 mg/kg/day) via intramuscular injection and ketoprofen (5 mg/kg/day) via intraperitoneal injection. The blood flow in the abdominal aorta was blocked temporarily and the tip of a transmitter for measuring pressure was inserted. The transmitter was sutured to the peritoneal cavity.

The rats were individually caged for 2 weeks to heal after the surgical cut. Then, the animals were placed in pairs in cages to reduce their isolation stress. The blood pressure was measured: before intraperitoneal administration of the compounds—time 0 min and 23 h thereafter in the 1st and the 15th day.

### Determination of the influence of the tested compound on the body’s temperature

The body temperature was measured using special loggers implanted subcutaneously (Star-Oddi, Island). Under general anesthesia (thiopental, 70 mg/kg, intraperitoneal), the loggers were inserted under the skin in the groin area and sutured with surgical thread. 24 h later, the baseline temperature was measured and then a starting compound was administered at a dose of 5 mg/kg b.w. The temperature measurement was performed for 24 h. The measurements were carried out every hour, with the first control 30 min after administration of compound. The test compound was given at 9:30 a.m. After 24 h, the loggers were removed and the body temperature data were read using a special set and software.

### Statistical analysis

Statistical calculations were carried out with the GraphPad Prism 6 program. Results are given as arithmetic means with a standard error of the mean. Statistical significance was calculated using a one-way ANOVA post hoc Dunnett’s Multiple Comparison Test, two-way ANOVA post hoc Bonferroni test or Multiple *t* test. Differences were considered statistically significant at: *P ≤ 0.05, **P ≤ 0.01, ***P ≤ 0.001.

### Drugs and compounds

Heparin was delivered by Polfa Warszawa S.A. (Warsaw, Poland), while thiopental sodium was from Sandoz International (France). Brimonidine, terazosin, phentolamine (reference compounds for intrinsic activity test) were purchased from Sigma-Aldrich (St. Louis, Mo, USA).

The tested compound; 1-(3-(4-(*o*-tolyl)piperazin-1-yl)propyl)pyrrolidin-2-one (EP-47) was synthesized in the Department of Physicochemical Drug Analysis, Chair of Pharmaceutical Chemistry, Faculty of Pharmacy, Jagiellonian University Medical College, Kraków, Poland.

## Results

**Fig. 1:**
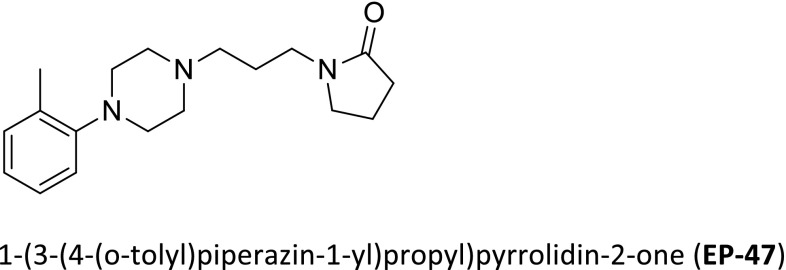
Formula for test compound: 1-(3-(4-(*o*-tolyl)piperazin-1-yl)propyl)pyrrolidin-2-one

Previously, we have identified a series of pyrrolidin-2-one derivatives bearing an arylpiperazine fragment, which exerted a strong binding to α-adrenoceptors [22]. Among 14 compounds, we selected a molecule, namely; 1-(3-(4-(*o*-tolyl)piperazin-1-yl)propyl)pyrrolidin-2-one (EP-47), which possess a strong affinity for α1- adrenoceptors with *K*_*i*_ = 95.5 ± 8.9 nM, and moderate affinity for α2-adrenoceptors *K*_*i*_ = 511.6 ± 34.8 nM. Figure 1 shows the chemical structure of the tested compound EP-47.

### Intrinsic activity

The tested compound showed significant antagonistic properties at the α_1B_-adrenoceptor (IC_50_ = 32.6 ± 0.69 nM) and weak at the α_1A_-adrenoceptor (IC_50_ = 42.5 ± 11.2 μM). It also interacted antagonistically with α_2A_-adrenoceptor (IC_50_ = 213 ± 32 nM). However, the functional response at the α_2B_-adrenoceptor was much weaker (IC_50_ = 2.07 ± 0.69 μM).

### The influence of the tested compound on the body weight of obese rats

**Fig. 2:**
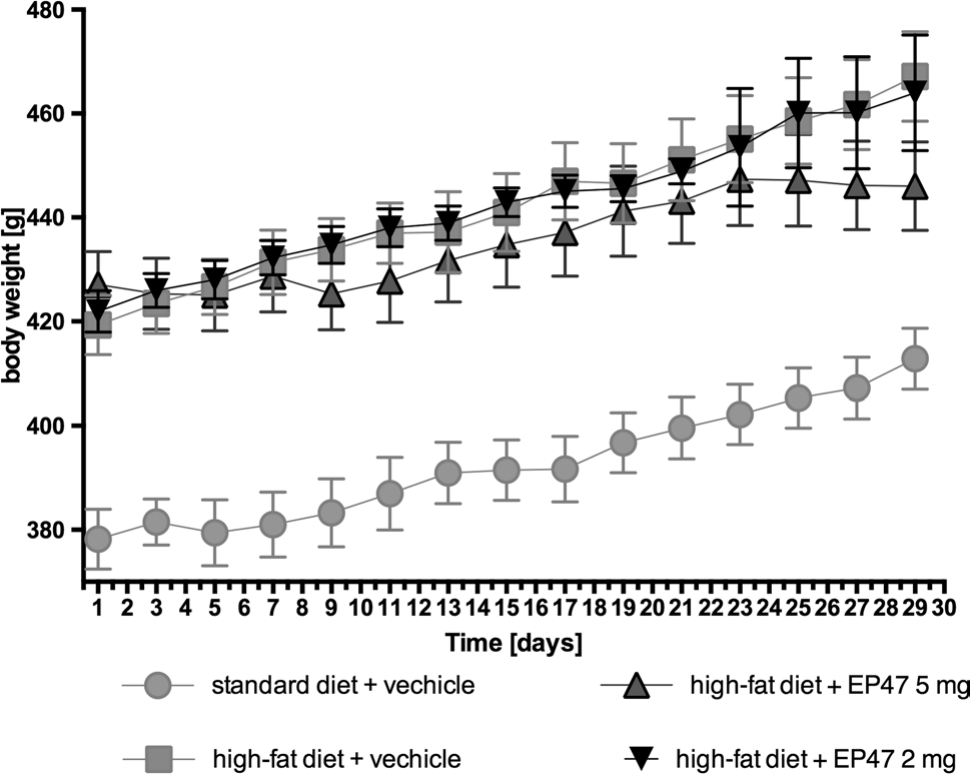
Effect of long-term administration of the α-adrenoceptor antagonist, 1-(3-(4-(*o*-tolyl)piperazin-1-yl)propyl)pyrrolidin-2-one, on body weight of diet-induced obese Wistar rats. The change in body weight in control (standard diet) and diet-induced obese Wistar rats, and diet-induced obese Wistar rats treated for 30 days with the tested compound. Results are mean ± SEM, *n* = 6. Multiple comparisons were by two-way ANOVA, post hoc Bonferroni test

The test compound administered at doses of 5 or 2 mg/kg b.w. intraperitoneally to rats fed on a high-fat feed did not cause a decrease in body weight gain compared to the obese rats (obesity control group = hight-fat feed + vechicle) [two-way ANOVA *F*_(32,315)_ = 0.2185, *P* = 0.9999]. The results are shown in Fig. 2.

### The influence of the tested compounds on the amount of peritoneal adipose tissue of diet-induced obese rats

**Fig. 3:**
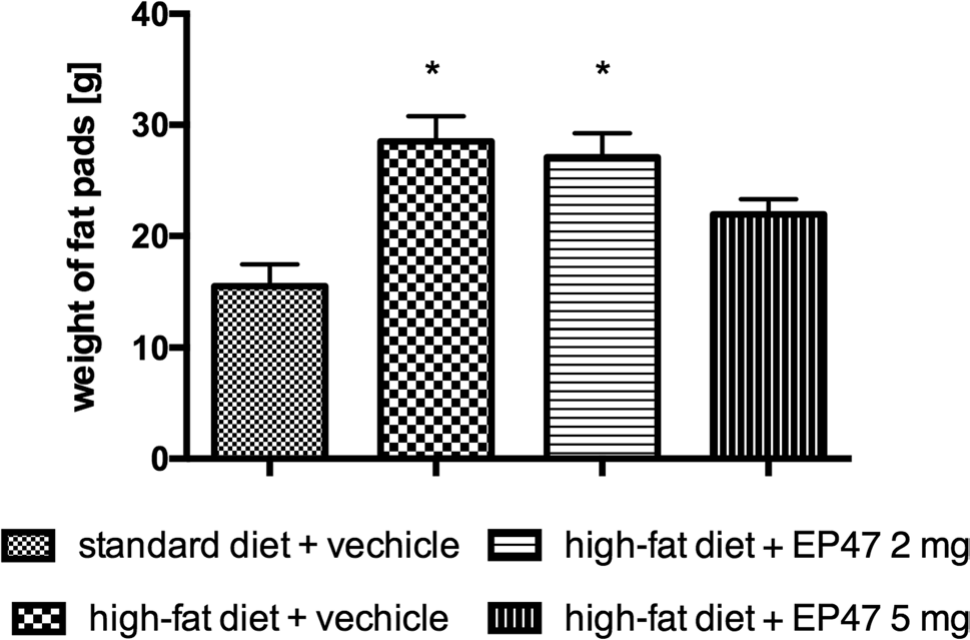
Weight of peritoneal fat after long-term administration of test compound in male Wistar rats in the obesity model. Results are mean ± SEM, *n* = 6. Multiple comparisons against the vehicle-treated control group were by two-way ANOVA, post hoc Bonferroni test. Significant differences are denoted by *P < 0.05

In groups receiving the test compound, the amount of adipose tissue pads was lower than that in obese rats, but the difference was not statistically significant. There was also no difference between the amount of adipose tissue in the treated and the control group [one-way ANOVA *F*_(3,21)_ = 7.521, *P* = 0.0013]. The results are shown in Fig. 3.

### Influence of the test compound on triglyceride and glucose levels in diet-induced obese rats

**Fig. 4:**
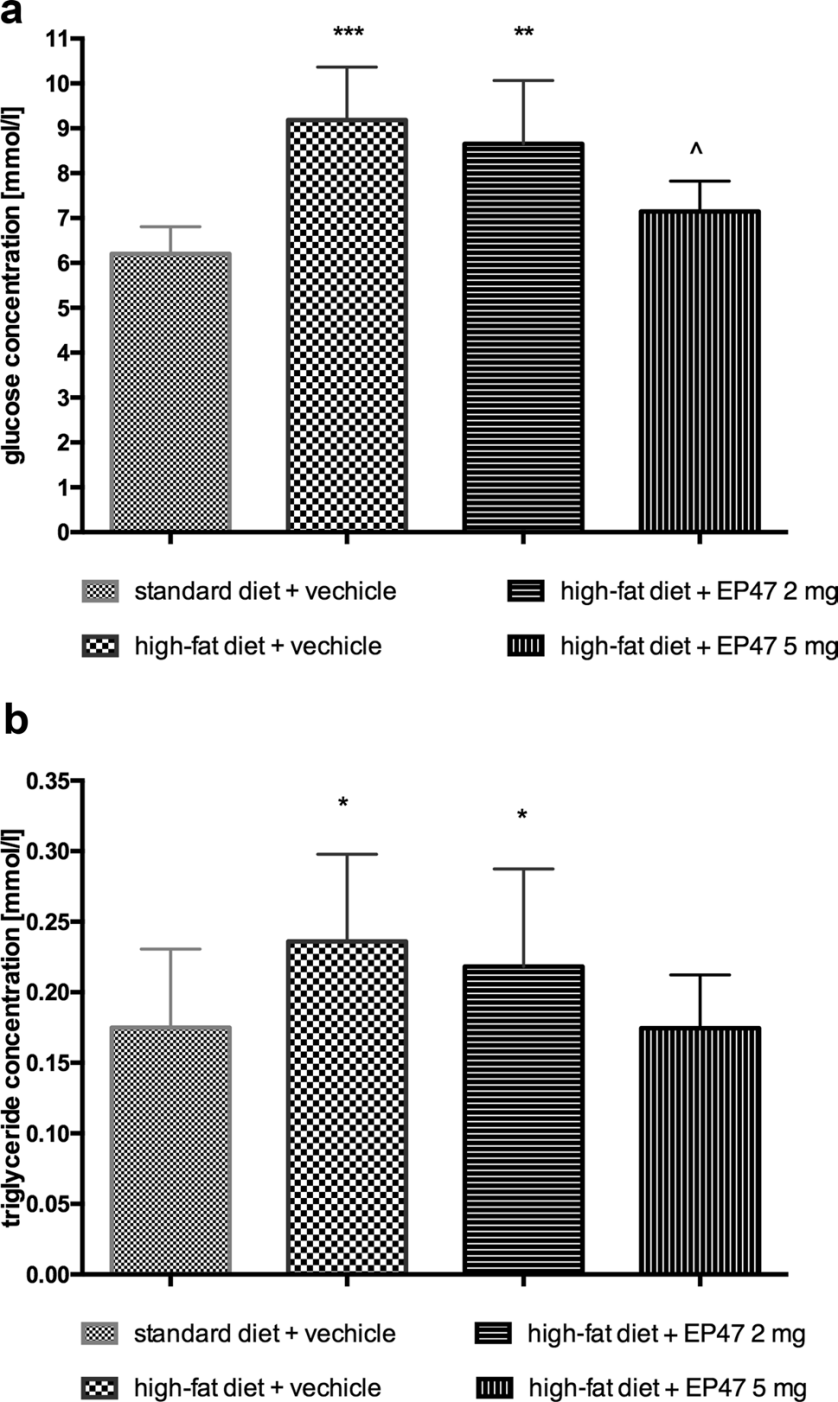
Effects of long-term administration of the test compound on plasma: glucose (**a**), triglyceride (**b**) levels in male Wistar rats in the obesity model. Results are mean ± SEM, *n* = 6. Concentrations in plasma: mmol/l. Comparisons against the vehicle-treated control group (asterisk) or against the vehicle-treated obesity control group (wedge) were by one-way ANOVA, post hoc Bonferroni test. Significant differences are denoted by ^*P* < 0.05, **P < 0.01

In animals treated with the test compound at a dose of 5 mg/kg b.w., intraperitoneal, significantly lower blood glucose level was observed as compared to the group of obese animals treated with water [one-way ANOVA *F*_(3,24)_ = 12.43, *P* = 0.0001]. The level of triglycerides was significantly lower in the group treated with the test compound compared to the level determined in the plasma of the obese control group [one-way ANOVA *F*_(3,24)_ = 5.153, *P* = 0.0068]. The results are presented in Fig. 4.

### The influence of the tested compound on locomotor activity after chronic treatment in diet-induced obese rats housed in pairs in home cages

**Fig. 5:**
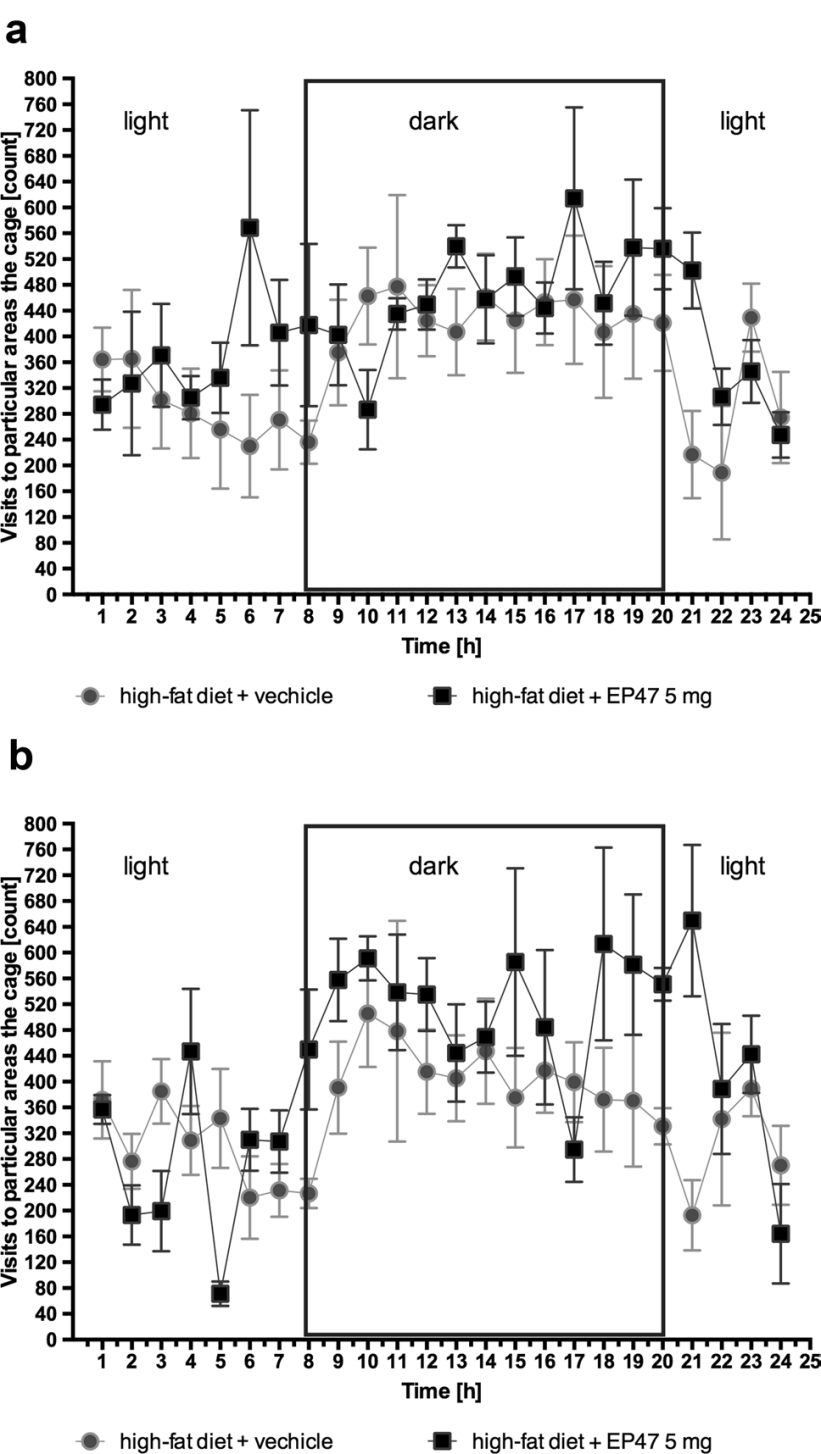
The influence of compound on locomotor activity after a single dose and chronic treatment in male Wistar rats in the obesity model. Locomotor Activity during 24 h period after treatment with test compound (5 mg/kg b.w.) or vehicle in diet-induced obese rats in 1st (**a**) and 29th (**b**) day of treatment. Activity is directly related to entrance to the various areas of the cage, *n* = 6 (Multiple *t* test)

The tested compound, at dose 5 mg/kg b.w., intraperitoneal, had no effect on locomotor activity after both single and chronic administration in obese rats. The results are presented in Fig. 5.

### The influence of the tested compound on blood pressure in anesthetized rats after single intraperitoneal administrations

**Fig. 6:**
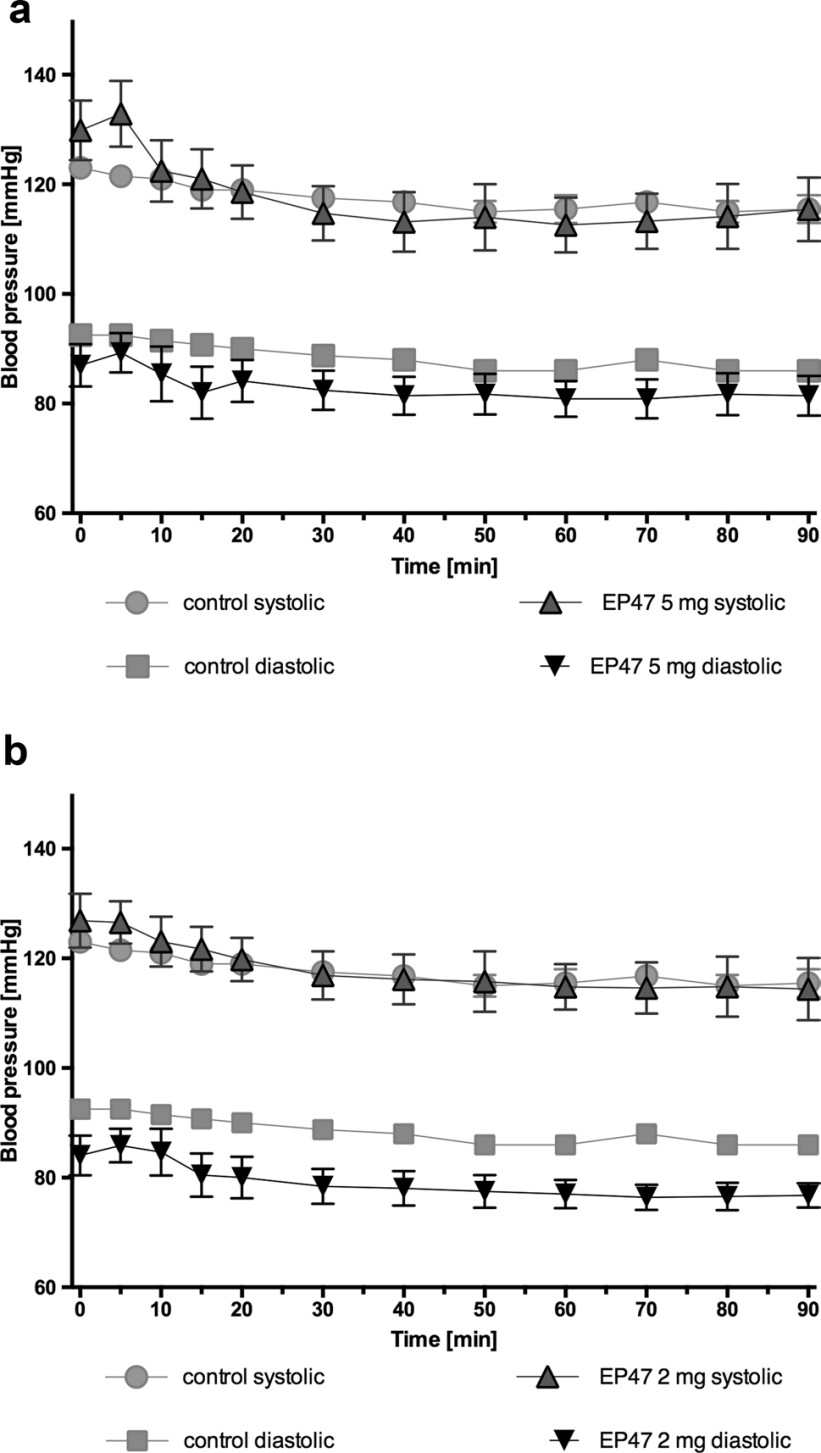
Changes in the blood pressure after single administration in anesthetized rats. Changes in systolic and diastolic blood pressure after a single, intraperitoneal administration of test compound at doses: **a** 5 mg/kg b.w. or **b** 2 mg/kg to rats fed a standard diet. Mean ± S.E.M.; *n* = 6 (two-way ANOVA test)

The tested compounds at a dose 5 mg/kg b.w., intraperitoneal, did not significantly affect blood pressure after a single administration [two-way ANOVA; systolic: *F*_(11,120)_ = 0.4434, *P* = 0.9332, diastolic: *F*_(11,120)_ = 0.09905, *P* = 0.9999]. The test compound at a dose 2 mg/kg b.w., intraperitoneal, did not significantly affect blood pressure after a single administration [two-way ANOVA; systolic: *F*_(11,108)_ = 0.01604, *P* = 0.9990, diastolic: *F*_(11,108)_ = 0.1519, *P* = 0.9993]. The results are shown in Fig. 6.

### The influence of the tested compound on the blood pressure in rats residing natural housing condition: the telemetric method

**Fig. 7:**
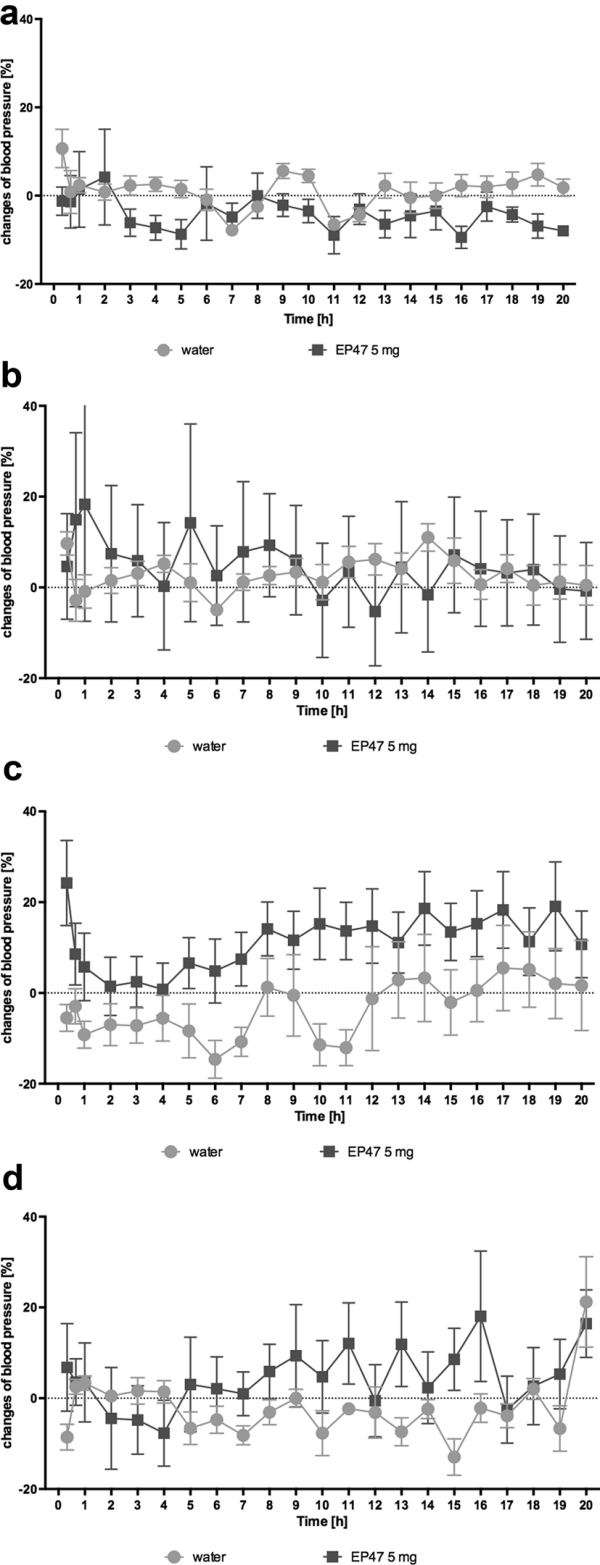
Changes in the blood pressure after administration in rats residing natural housing condition—telemetric methods. Changes in systolic (**a**) and diastolic (**b**) blood pressure after a single intraperitoneal administration of test compound at doses: 5 mg/kg b.w. to rats fed a standard diet. Changes in systolic (**c**) and diastolic (**d**) blood pressure after 15th times intraperitoneal administration of test compound at doses: 5 mg/kg b.w. to rats fed a standard diet. Percentage of pressure changes compared to the pressure measured before administration of the test compound. Mean ± S.E.M.; *n* = 6 (two-way ANOVA test)

After single and 15th intraperitoneal administration of the tested compound at a dose of 5 mg/kg b.w. to rats residing in natural housing conditions, there was no significant changes in systolic and diastolic pressure, compared with rats that received water. The results are presented in Fig. 7.

### Influence of the test compound on the body temperature after a single administration

**Fig. 8:**
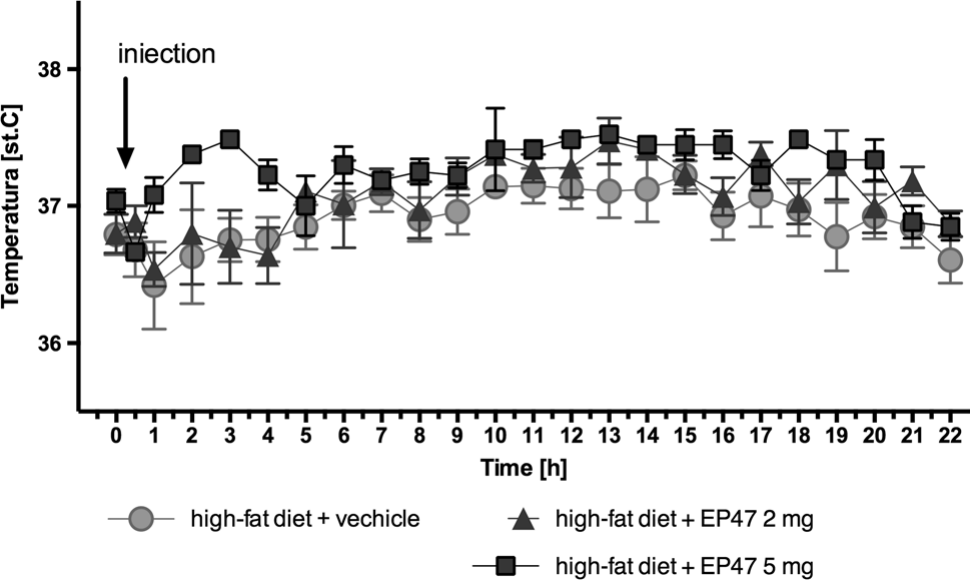
The influence of test compound on temperature after a single dose. The changes in temperature after a single, intraperitoneal administration of compound or vehicle (0.3 ml) to non-obese rats; time 0 = 0.5 h before administration of compound (9.00 a.m.); Mean ± S.E.M.; *n* = 6; (two-way ANOVA test)

The test compound did not influence significantly the body temperature for 22 h after treatment in obese rats after one administration [two-way ANOVA; *F*_(46,264)_ = 0.6823, *P* = 0.9407]. Results are shown in Fig. 8.

## Discussion

Imbalanced lipid–carbohydrate homeostasis, the obesity, and the hypertension are the key components of the metabolic syndrome. Due to the vast distribution of the adrenoceptors in the body, and its impact on the blood pressure, metabolism of triglycerides and glucose, the α-adrenoceptor antagonists may be useful in the treatment of many types of disorders. In this paper, we investigated the effect of the pyrrolidin-2-one derivative on the body weight, hyperglycemia, hypertriglyceridemia and blood pressure in the animal model of obesity induced by a high-fat diet, measuring additionally the spontaneous activity and the temperature. The tested compound; 1-(3-(4-(*o*-tolyl)piperazin-1-yl)propyl)pyrrolidin-2-one (EP-47) was selected from the library of previously synthesized and described structures, considering its non-selective affinity for the adrenoceptors with a preference towards the α_1_-adrenoceptors [22]. We extended the pharmacological profile characteristics of the selected molecule, by additional intrinistic activity assays, which revealed that the EP-47 is a potent antagonist of α_1B_ and α_2A_-adrenoceptors. While designing the present study, we considered the recent literature data regarding the ability of α_2A_-adrenoceptor antagonists to indirectly induce the weight loss in animal models of obesity [18, 20, 23]. We postulated that, since the tested compound is a α_2_-adrenoceptor antagonist, it is able to increase the release of noradrenaline, which stimulates the β-adrenoceptors located in adipocytes, and this could lead to increased lipolysis, thermogenesis and consequently to the weight loss. Therefore, we measured the influence on the body weight in the animal model of obesity induced by a high-fat diet, after the chronic administration of the tested compound. However, despite expected activity, the tested compound did not reduce the body weight in obese animals.

During the present study, in the animal model of obesity induced with a fatty diet, we observed a statistically significant increase in the glucose or triglyceride levels in the collected rat plasma [18, 20]. Since the tested compound exerts also antagonistic properties towards the α_1_-adreneoceptor, it could potentially improve the impaired lipid profile and carbohydrate metabolism [9, 10]. As a proof of concept, we observed a statistically significant reduction in the glucose and triglyceride levels in animals treated with the tested compound. One can presume that this effect is associated with the α_1B_-adrenoceptor inactivation, as the tested compound potently inhibited this particular subtype, while weakly affecting the α_1A_-subtype. However, in the literature there are no detailed studies which could clearly explain which α_1_-adrenoeptor subtype, contributes to the carbohydrate and lipid profile improvement. Therefore, we believe that the obtained results from our studies are particularly valuable.

On the other hand, the ability to balance the plasma glucose levels by the tested compound may also be associated with the inhibition of α_2A_-adrenoceptors. The α_2A_-adrenoceptors are widely distributed in the body, and are also present in the pancreatic islet β-cells [24]. During physiological conditions, catecholamines that act on the postsynaptic α_2A_-adrenoceptors located on the pancreatic islet β-cells, inhibit the insulin secretion and consequently increase the blood glucose levels [25]. Thus, the reduction of the α_2A_-adrenoceptor activity may facilitate the insulin secretion from the pancreatic islets and, as a result, reduce elevated glucose levels [26]. Previous studies demonstrated an improved insulin secretion in the presence of the non-selective α_2_-adrenoceptor antagonist—yohimbine in diabetic patients [27], as well as the reduction of the glucose level by yohimbine in animal model of obesity induced by a high-fat diet [20] and in ob/ob mice (results submitted for publication). It would be interesting to consider the use of the α_2A_-adrenoceptor antagonist for the prevention or the treatment of the stress-induced hyperglycemia and associated with it; morbidity and mortality. Currently, there is no α_2A_-adrenoceptor antagonist in clinical use and the side-effect profile of yohimbine is unacceptable to be considered as a potential treatment of the hyperglycemia.

In addition, the modest reduction of peritoneal fat piles was observed in the tested animals. The body fat mass was lower than the mass of adipose tissue in untreated obese rats. However, the body fat mass was higher than in the non-obese control group. These results correlate with the lower levels of triglycerides in the plasma of the treated animals as compared to untreated animals. The observed reduction of the lipid and glucose levels after administration of the tested compound over a 30-day period, may suggest that its prolonged use could eventually lead to the reduction of the body weight in the test animals, as a result of a balanced lipid and carbohydrate economy which is often an important first step to weight loss.

The effect on the spontaneous activity of the animals was evaluated additionally. The modern equipment available in our laboratory enables us to precisely determinate the location of an animal in the cage. Therefore, for the assessment of the spontaneous activity, we used the same group of animals for the measurement of body weight in obese animals. The changes in spontaneous locomotor activity could be a possible indicator of side effects of the tested compound [28]. In the present study, the tested compound did not significantly affect spontaneous activity, thus indicating the absence of adverse behavioral reactions.

The temperature regulation in the brown adipocyte tissue is mediated by the adrenergic system. Adipose tissue is highly innervated by the sympathetic nerve system, which becomes activated when exposed to cold and food. Since the activation of α_2_- and β_3_-adrenoceptors causes an opposite effect to each other, the α_2_/β_3_-adrenoreceptors ratio is very important, not only in the thermogenesis, but also in the control of the lipolysis in the white adipose tissue [29]. It has been shown that, the body temperature changes correlate with the thermogenesis [30] and α_2A_-adrenoceptor antagonists are able to increase the thermogenesis, which consequently reduces the body weight [31]. The body is forced to use energy to recover the body temperature back to normal. In the present study, the tested compound did not significantly affect the body temperature of the animals in the natural habitat, despite its ability to inhibit the α_2A_-adrenoceptor. The tested compound exhibits also antagonistic properties at the α_1B_-adrenoceptor. However, according to the literature data, both non-selective α_1_-adrenoceptor antagonists, such as prazosin, as well as selective α_1A_-adrenoceptor antagonists—RS100329 or cyclazosin—α_1B_-adrenoceptor antagonist do not affect the body temperature [32]. The obtained results are correlated with the lack of impact on body mass, but the mechanism of action remains unclear. Nevertheless, the application of an innovative telemetric system to measure the body temperature ensured of getting credible results, which were obtained during the measurement without unnecessary interference in the animal behavior that could lead to the measurement-associated stress.

The tested compound did not impair the blood pressure in normotensive animals at the tested dose. We have not observed a significant increase in the blood pressure, triggered by excessive noradrenalin release [33], which is a characteristic for α_2A_-adrenoceptor antagonists [20]. Neither have we observed a sharp drop in the blood pressure due to a rapid vasodilatation caused by the α_1B_-adrenoceptor antagonists [8, 34]. The obtained results are likely related to the interesting pharmacological profile of the tested compound, which potently blocks these two subtypes of the adrenergic receptor. This sort of action can be specified as the functional antagonism in terms of the blood pressure regulation. The obtained results seem to be particulary interesting, considering that there are many clinical cases of patients with disturbed lipid and carbohydrate profile, but without hypertension. Additionally, there are also cases when high blood pressure is the main issue. However, it can be effectively treated with first-line drugs in this indication, such as metabolically neutral angiotensin converting enzyme inhibitors or beta-blockers that are metabolically adverse. This group of patients may need an add-on therapy with a drug, which improves the carbohydrate and lipid profiles, without interfering with blood pressure.

Although the obtained results are interesting regarding the potential treatment of patients with the metabolic syndrome, authors have recognized the following limitation of the conducted studies:

1. The insulin levels were not determined at the end of the experiment. Such analysis would be valuable, since α_2_-adrenoceptor antagonists might favorably modulate the pancreatic function and the abnormal insulin secretion [26]. The determination of the insulin level would clarify whether the reduced blood glucose level observed after the administration of the test compound, resulted from the insulin release mediated via α_2A_-adrenoceptors. This issue will be considered in our future studies.
2. The determination of circulating catecholamine levels at the end of the study would be valuable, since α_2_-adrenoceptor antagonists enhance the release of catecholamines [11] and thus could induce potential negative cardiovascular effects. Circulating catecholamines bind to the β-adrenoceptors, and a long-term cardiac stimulation of the β-adrenoceptor causes cardiac hypertrophy and dysfunction. The cardiac remodeling is also a compensation process, in which the expression of β-adrenoceptors is down-regulated to prevent the receptor from over-activation in the heart [35]. Adrenergic receptors are readily susceptible to changes in the density and sensitivity. Depending on the condition, constant stimulation or blockade may lead to its down or up-regulation. A compound which is able to enhance the release of catecholamines through the stimulation of the beta-adrenergic receptors in the heart, may initially induce a desirable inotropic effect. However, the long-term stimulation of the β-adrenergic receptors, may lead to a decrease in receptor density and sensitivity, and thus adversely affect the existent heart failure. During the heart failure, the amount and sensitivity of adrenoceptors is pathologically reduced, thus chronic administration of an α_2_-adrenoceptor antagonist that increases the release of catecholamines seems to be a relatively unfavorable reaction. Therefore, one may wish to determinate additional changes in the adrenoceptors density in the heart, after chronic administration of the test compound.
3. Although the effect of the tested compound on the blood pressure using the telemetric method has been determinated, it would be interesting additionally to evaluate the direct influence of the tested compound on vascular contractility. This study would reveal whether the test compound is able to dilate the blood vessels by blocking the α_1_-adrenoceptors. Additionally, one may wish to evaluate the influence on the blood pressure in the animal model of hypertension.
4. Since α_1_-adrenoceptor antagonist enhances ischaemia-induced neo-angiogenesis independently of vasodilatation [36], it would be interesting to determine the influence of the tested compound on the vascular endothelium and the angiogenesis process.

Nevertheless, all the issues listed above will be addressed in our future studies.

## Conclusion

The present study indicated that a non-selective α_1B_-/α_2A_-adrenoceptor antagonist; 1-(3-(4-(*o*-tolyl)piperazin-1-yl)propyl)pyrrolidin-2-one effectively reduced the elevated glucose and triglyceride levels in the animal model of obesity induced by a high-fat diet. The tested compound only moderately reduced the amount of peritoneal body fat. It neither affected the body temperature nor caused any disturbance in the spontaneous activity and the blood pressure. The lack of influence on blood pressure is particulary significant, since currently there are no α_2A_-adrenolytic agents available in clinical use which would not impair blood pressure. Therefore, the search for novel molecules with a similar activity profile to the tested compound, seems to be a promising strategy regarding the potential treatment of hyperglycemia, hyperlipidemia induced by a high-fat diet. Maintaining stable euglycemia is one of the aims of an effective therapy of the metabolic syndrome as well as acute cardiac conditions, such as myocardial infarction, as it may significantly reduce the risk of cardiac complications in patients.

We believe that non-selective α_1B_-/α_2A_-adrenoceptor antagonists might have a potential in the treatment of the metabolic syndrome, therefore 1-(3-(4-(*o*-tolyl)piperazin-1-yl)propyl)pyrrolidin-2-one requires further extended studies to explore its full pharmacological profile.
